# The protective value of a defensive display varies with the experience of wild predators

**DOI:** 10.1038/s41598-018-36995-9

**Published:** 2019-01-24

**Authors:** Kate D. L. Umbers, Thomas E. White, Sebastiano De Bona, Tonya Haff, Julia Ryeland, Eleanor Drinkwater, Johanna Mappes

**Affiliations:** 1 School of Science & Health, Western Sydney University, Locked Bag 1797, Penrith, NSW 2751 Australia; 2 Hawkesbury Institute for the Environment, Western Sydney University, Locked Bag 1797, Penrith, NSW 2751 Australia; 3 School of Life and Environmental Sciences, University of Sydney, Sydney, NSW Australia; 4 Centre of Excellence in Biological Interactions, Department of Biological and Environmental Sciences, University of Jyväskylä, Jyväskylä, Finland; 5 Department of Zoology, Cambridge University, Cambridge, UK

## Abstract

Predation has driven the evolution of diverse adaptations for defence among prey, and one striking example is the deimatic display. While such displays can resemble, or indeed co-occur with, aposematic ‘warning’ signals, theory suggests deimatic displays may function independently of predator learning. The survival value of deimatic displays against wild predators has not been tested before. Here we used the mountain katydid *Acripeza reticulata* to test the efficacy of a putative deimatic display in the wild. Mountain katydids have a complex defence strategy; they are camouflaged at rest, but reveal a striking red-, blue-, and black-banded abdomen when attacked. We presented live katydids to sympatric (experienced) and allopatric (naive) natural predators, the Australian magpie *Cracticus tibicen*, and observed bird reactions and katydid behaviors and survival during repeated interactions. The efficacy of the katydids’ defence differed with predator experience. Their survival was greatest when faced with naïve predators, which provided clear evidence of the protective value of the display. In contrast, katydid survival was consistently less likely when facing experienced predators. Our results suggest that sympatric predators have learned to attack and consume mountain katydids despite their complex defense, and that their post-attack display can be an effective deterrent, particularly against naïve predators. These results suggest that deimatism does not require predator learning to afford protection, but that a predator can learn to expect the display and subsequently avoid it or ignore it. That sympatric predators learn to ignore the defense is a possible explanation for the mountain katydid’s counter-intuitive behavior of revealing warning colors only after tactile stimuli from predator attack.

## Introduction

The study of visually conspicuous defensive signals has driven the development of fundamental evolutionary theory in predator-prey interactions^1,2^, mimicry^3–5^, cognition^6^ and speciation^3,7,8^. Particular progress has arisen from work on aposematic systems, in which prey advertise their unpalatability to predators via static (‘always on’) colour patterns^9^. Defensive displays, however, can include multiple elements such as a sudden transition between camouflage and aposematism, or the dynamic presentation of an otherwise concealed defence^10–16^. It has been suggested that a sudden transition could constitute a deimatic component of such defensive displays, but empirical data required to test this idea are scarce^17–23^. There are likely profound differences between the aposematic and the deimatic components, with respect to the mechanisms by which they deter predators (see below) and these differences have ecological and evolutionary implications, for which there is a paucity of data^17,23^.

The adaptive value of defensive signals lies in their ability to interrupt the predation sequence^24^. In the case of aposematism, naïve predators must initially learn to associate prey signals with defenses - unless they exploit pre-existing biases - which reduces the likelihood of future encounters escalating to an attack^25,26^. The protective value of an aposematic signal thus tends to increase as a function of predator experience. It has been predicted that due to its sudden transition, deimatism does not require predators to accumulate experience, and therefore that its protective value is greater against inexperienced predators compared to that of aposematism^17^. If deimatic prey do not deploy a protective secondary defence such as weaponry or chemical defenses, the efficacy of their display could decrease as a function of predator experience, if predators learn to suppress their aversive response^27^. Alternatively, this effect may be ameliorated by the presence of further co-occurring defences, such as an aposematic element combined with a deimatic element.

Here we tested the role of predator experience in shaping the protective value of a defensive, putatively deimatic, display in the wild. We examined the responses of a known avian predator to an insect in assays designed to quantify both the absolute and relative protective value of the display across natural variation in predator experience. If the display exploits a reflexive response in the predator, which does not require learned aversion, we predicted that upon first presentation naïve predators should be less likely to attack, kill, and eat the prey than experienced predators. If, in contrast, the display is solely reliant on the formation of learning rules in a manner akin to aposematism, then its protective value should be greatest against experienced predators. Further, we predicted that experienced predators would respond consistently between their first and second encounter with katydids, while naïve predators would attack the unknown prey on their first interaction and, once startled, abandon the hunt. On their second encounter, we expected previously naïve predators to either: (a) avoid the insect, if they learned to associate the display (and/or co-occurring defence) with unprofitability; or (b) attack the insect, if they learn to expect the display and the insect is sufficiently profitable prey.

## Methods

### Predator habituation and prey handling

**Figure 1:**
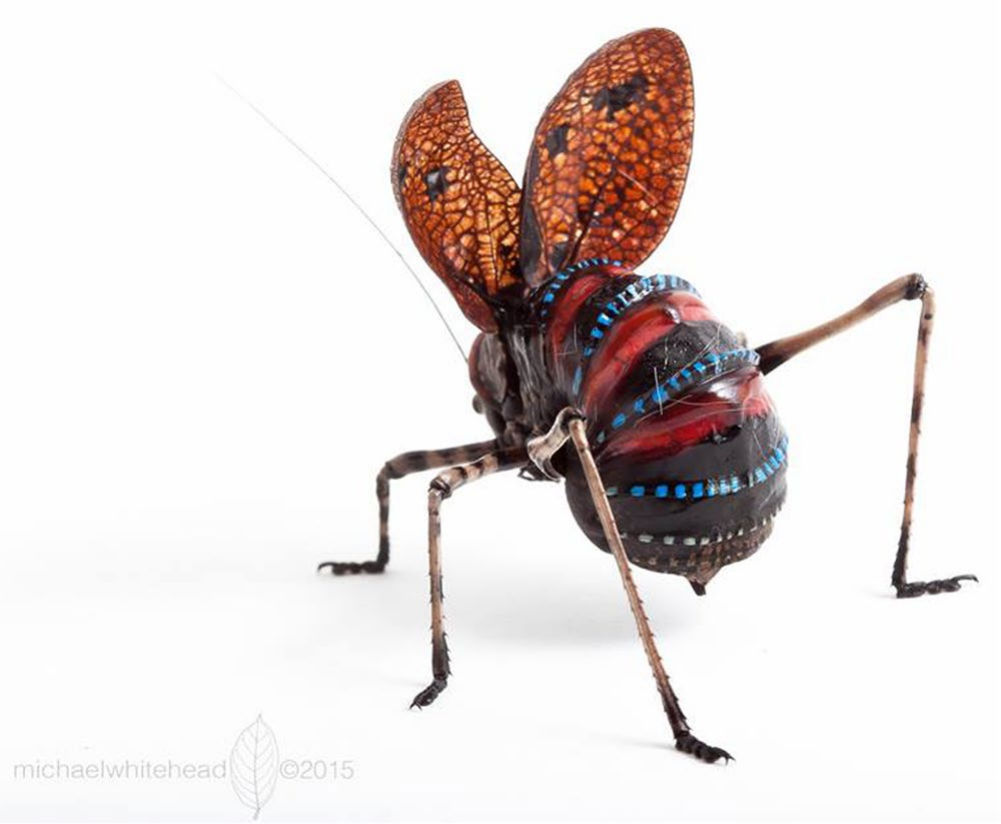
Photograph of an adult female mountain katydid performing her display (Credit: Michael R. Whitehead).

The mountain katydid (*Acripeza reticulata*, Guérin-Méneville 1832) is a large, putatively chemically defended orthopteran native to the montane regions of south-eastern Australia^28,29^. Mountain katydids have tough cover wings (tegmina) and are cryptic at rest, but reveal a striking red-, blue-, and black-banded abdominal pattern when attacked (Fig. 1). Recent behavioral assays with mountain katydids showed that they perform a defensive display after receiving a tactile stimulus (i.e. an attack by a predator), but not in response to visual stimuli^30^. The explanation for this late defensive response is currently unclear. Potentially, the katydids lack the ability to reliably detect visual cues, or perhaps visual cues are detected, but simply ignored because they are too unreliable. Displays are held between 30 s and 300 s^30^.

In addition to their visual display, mountain katydids regurgitate their crop contents, and exude a bitter tasting liquid from their abdomen. Both are likely rich in alkaloids derived from their *Senecio* host plants (Umbers *et al*. unpublished data) but the effect of those alkaloids on predators is currently unknown. The Australian magpie, *Cracticus tibicen*, is a known primary threat to mountain katydids in the wild, and we therefore selected them as our focal predator^31^. Australian magpies are easily habituated to humans^32,33^, common both within and outside of the mountain katydid’s range^29^, and have a generalist omnivorous diet that includes ground dwelling insects^32^. Magpies hold small (~2 km^2^), distinct, stable, year-round territories that rarely overlap with conspecifics^32^. Their plumage is highly variable and sexually dimorphic, allowing the reliable identification of individuals in a small family group. By never visiting a territory more than once we further ensured independence of trials. We conducted trials with Australian magpies sympatric to mountain katydids in human-inhabited areas of Kosciuszko National Park, NSW (24 locations bounded by Tom Groggin: −36.591656, 148.086858; Bullocks Flat, −36.444316, 148.441767; Geehi: −36.330308, 148.242039), and with allopatric magpies throughout the suburbs of Sydney (42 locations in Sydney bounded by Richmond: −33.609777, 150.755583; La Perouse: −33.992104, 151.247570 and Brisbane Water: −33.478759, 151.313832, all areas outside of the mountain katydid’s range). Though the genetic structure of groups and relatedness of individual magpies within each location is unknown, our current understanding of their ecology (as above) strongly suggests that we sampled from numerous populations within these broad zones of ‘sympatry’ and ‘allopatry’. As detailed below, we also include location effects in our statistical analyses, thus controlling for any possible cultural transmission and social learning among family groups. We targeted magpies in Kosciuszko National Park that held territories within or close to ski resorts (~500 year-round residents), camping grounds, and caravan parks, both to ensure that they were habituated to humans and that they had access to ample food. The magpie populations within both Kosciuszko and Sydney often displayed ‘urbanised behaviours’ such as foraging in bins, picnic tables, and taking food from people. Through this approach, the birds from Sydney and the birds from Kosciuszko were comparable, despite being wild, free ranging predators. In many of the sympatric magpie territories, mountain katydids have been seen by us (KU, SDB), could be heard calling, and/or their *Senecio spp*. host plants were visible when experiments were taking place. Thus, we expect populations of sympatric magpies had experience with mountain katydids. The sympatric and allopatric ranges are separated by approximately 400 km. We ran the experiment concurrently across both sympatric and allopatric magpies through March and April, 2016. We habituated magpies using small pieces of cheese as rewards before the commencement of trials to enable us to approach close enough to gently present stimuli.

We collected 264 female mountain katydids from patches of *Senecio gunnii* and *S. linarifolius* in Kosciuszko National Park in March and April 2016. Mountain katydids are sexually dimorphic and females were chosen because, unlike males, they cannot fly and have a very large striking blue and red display (Video S1). We housed individuals in mesh enclosures no smaller than 300 mm × 200 mm × 200 mm. We provided mountain katydids with *ad libitum* access to water and their preferred native, alkaloid-rich food plants *S. gunnii* and *S. pinnatifolius* (Umbers *et al*. unpublished data), thereby ensuring the maintenance of their putative chemical defence and similar levels across all katydids used in the experiment. No katydid was held for longer than seven days before being used in an experimental trial.

### Treatment protocols

We assigned habituated predators to one of three treatments, defined by the focal stimulus being presented. For a given trial, we presented each individual predator with a stimulus from their allocated treatment twice, with an interval of two minutes between presentations. We minimized the potential for social learning by luring away all individuals in the immediate vicinity of the test subject (ca. 10 m) using small pieces of cheese as bait before treatment presentation. The test stimulus was one of: (1) a mountain katydid (2.38 ± 0.52 g); (2) a palatable orthopteran (local grasshopper, *Kosciuscola tristis* (~0.5 g), a common insect in sympatric locations; or house crickets, *Acheta domesticus* (~0.5 g), a common pest insect in allopatric locations); (3) an inedible stimulus, to control for the conditioning of wild birds to humans as sources of food (a soft grey plasticine ball, similar in size to the katydid). Unpalatability is always relative, so we chose an inedible control and a palatable control with the expectation that the katydid would be more acceptable than the inedible control and less acceptable than the palatable control. The inedible stimulus also provided a negative control that was undeniably less profitable than a katydid. Among sympatric birds, we ran trials in the fixed order outlined above (katydid, cricket, ball) to minimise bias toward individual predators. For the allopatric birds we ran the trials in a slightly different order (ball, katydid, cricket, katydid), to ensure that the katydids we relocated to Sydney were not held for longer than seven days.

For a given trial, we lured individual magpies within two meters of the experimenters using small pieces of cheese, identical to that with which birds were habituated. Once the bird was within this range for 30 seconds, we gently tossed the assigned stimulus within one meter. The background against which the katydid was presented varied depending on the location of the magpie being tested, however the obvious movement of the stimulus as it was presented was enough for the bird to detect the prey item each time. The backgrounds consisted of grass, cement, or asphalt in both locations. The bird was then allowed to interact with the stimulus until this was attacked and rejected, killed, eaten or ignored. Once the interaction was over, we removed the remaining stimulus if it was not consumed and waited two minutes before presenting a second stimulus. As is a risk when working with wild predators, not all birds stayed to receive the second presentation (ca. 9% did not complete the two presentations), so we excluded these trials from our analyses. We also excluded trials in which magpies failed to notice the stimulus at the beginning of either presentation (ca. 4%), and a single trial in which a naïve bird avoided the katydid before seeing their display, with a final total of 163 complete trials.

### Scoring magpie behavior

We scored magpie behavior at the time of the presentation (JR, ED, KU, GM), and afterwards from video footage collected during the trials (Video S1). We evaluated each predatory encounter on an ordinal scale of one to five, which corresponded to whether a magpie: (1) saw the stimulus (quickly turned head in the direction of the stimulus and then held a sustained pause), (2) approached the stimulus (walked towards), (3) attacked the stimulus by making physical contact using its beak, (4) killed the stimulus, usually by repeatedly hitting it against a hard surface and/or tearing pieces off it, and (5) consumed the stimulus. If the predator attacked and rejected the prey, its score was 3. All categories were easy to observe during the magpies’ behavior except for kill, which was assessed once the magpie had stopped interacting with the stimulus.

### Statistical analyses

We used mixed-model ordinal logistic regression to test the effects of treatments, experience, and presentation number on the probabilities of the behavioral escalation of magpies during predation^34^. We modelled the responses of magpies coded on a scale of one to five (detailed above) as an ordinal response variable, and included treatment (inedible, control/palatable control/katydid), presentation number (one/two) and predator experience (sympatric/allopatric) as main effects, with location and magpie-ID as random effects. We used log-likelihood ratio χ^2^ to test the significance of the overall model and individual predictors. All analyses were run in R^35^, primarily using the clmm function in the package ordinal (v2018.4-19^36,37^). All p-values quoted are two-tailed.

### Ethics Statement

All experiments were conducted in accordance with relevant guidelines and regulations under ethics permit number: AE14/35 from the University of Wollongong.

## Results

**Figure 2:**
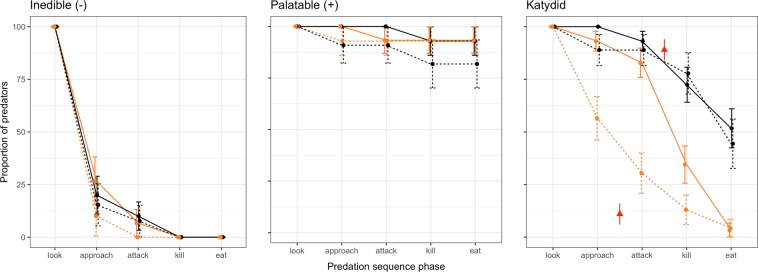
The proportion of predators (±s.e.) in control and experimental treatments that escalate their response through each stage of the predation sequence. Sympatric (experienced) predators are denoted by black and allopatric (naïve) denoted by orange lines and points, while presentation number is indicated by solid (first) and dashed (second) lines. Red points in the katydid treatment identify the mean proportion of katydids (±s.e., pooled in the absence of a between-population difference) that displayed prior to and immediately following the initial attack by avian predators. The category ‘kill’ is retained in the plasticine ball figure to standardize presentation of the data.

In total, we presented stimuli to 163 different, free-ranging, wild Australian magpies, two of the same stimuli per bird. Of those sympatric to mountain katydids, we presented 29 magpies with plasticine balls, 24 magpies with local grasshoppers and 29 magpies with mountain katydids. To allopatric magpies we presented 21 magpies with the plasticine ball, 23 magpies with house crickets and 37 magpies with mountain katydids. Any differences in the sample sizes for each treatment were due to ensuring that we used as many katydids as quickly as possible so we did not hold them for more than 7 days. The responses of magpies differed between experimental treatments, individual stimulus presentations, and the extent of prior experience with katydids as estimated by sympatry or allopatry (GLMM three-way interaction χ^2^ = 15.59, df = 6, *P* = 0.016; Fig. 2; Video S1). Birds that were presented palatable insects (grasshoppers and crickets) attacked and ate them. The responses of magpies to katydids varied with long-term experience. Allopatric magpies, naïve to the mountain katydid, were less likely to escalate their encounters than experienced, sympatric magpies (Fig. 2c). We also found evidence for a differential effect of presentation between predators; allopatric, but not sympatric, predators were less likely to escalate their encounters upon secondary presentation of mountain katydids (Fig. 2; Table 1). There was no discernible difference in the overall responses of predators to the inedible control between experienced (sympatric) and naïve (allopatric) predators, or between presentations (Table 1).

**Table 1:** Model coefficients (Est.), standard errors (SE), and 95% confidence intervals (CI) for magpie predation escalation (on an ordinal scale of 1–5) as a function of experimental treatment (palatable control/inedible control/mountain katydid), experience (allopatric/sympatric) and stimulus presentation (one/two).

	Est.	SE	lower CI	upper CI
**Treat (palatable)**	**9.74**	**1.77**	**6.26**	**13.22**
**Treat (katydid)**	**4.18**	**0.95**	**2.32**	**6.04**
Treat (inedible) x Experience (sympatric)	−0.23	0.95	−2.09	1.63
Treat (palatable) x Experience (sympatric)	−0.01	1.72	−3.36	3.37
**Treat (katydid) x Experience (sympatric)**	**2.11**	**0.60**	**0.92**	**3.30**
Treat (inedible) x Experience (allopatric) x Presentation	−1.26	1.29	−3.79	−1.27
Treat (palatable) x Experience (allopatric) x Presentation	−0.21	1.61	−3.37	2.95
**Treat (katydid) x Experience (allopatric) x Presentation**	**−2.05**	**0.58**	**−3.20**	**−0.91**
Treat (inedible) x Experience (sympatric) x Presentation	−0.57	1.09	−2.70	1.56
Treat (palatable) x Experience (sympatric) x Presentation	−0.77	1.63	−3.97	2.41
Treat (katydid) x Experience (sympatric) x Presentation	−0.51	0.65	−1.78	0.76

Bolded parameters indicate significance at α = 0.05.

## Discussion

The efficacy of many defensive strategies arises from predators learning to associate prey defenses with corresponding signals^38^. In experiments with predators of contrasting experience, we found clear evidence supporting our expectation that the mountain katydid’s defensive display does indeed have a protective value (Fig. 2). Thus, despite requiring tactile stimuli to elicit the display, the display can save the katydid’s life, and this is most likely if the predator is naïve. However, our data suggest that the degree of protection conferred, and the point during a predation event at which the benefits manifested, varied with predator experience (Fig. 2c). Our data confirm that mountain katydids wait until they have been physically attacked before they perform their display, hinting at the importance of the putative camouflage (non-displaying) phase for survival^30^.

In addition to demonstrating the effectiveness of the katydid’s display, our data support our prediction that experienced predators respond consistently toward katydids between the first and second presentations. Unexpectedly, however, rather than avoiding these seemingly well-defended insects, experienced (sympatric) predators were more likely to attack, kill and consume katydids than naïve predators. This indicates that katydids are in fact profitable prey for experienced predators, or at least relatively so, because (a) they can physiologically tolerate or behaviorally remove their chemical defense^31^, (b) the toxin load is worth the trade-off^39,40^, or (c) because sympatric predators learned to ignore the katydids’ defences over many encounters, suggesting that they are not as well-defended as they seem^27^.

On first presentation, katydid defenses were far more effective against naïve, allopatric predators, than experienced, sympatric predators. This result is consistent with the hypothesis that deimatic displays do not require predator learning, in contrast to what is generally expected for aposematism^1,11,17,24,25^. The katydid’s effective defense against naïve predators is unlikely to be explained by neophobia, because the majority of magpies approached and attacked the katydids while the katydids were in their camouflage phase. Upon second presentation, however, allopatric predators avoided katydids, seeming to have learned to avoid them from their first experience.

Sympatric and allopatric predators reacted to the presentation of mountain katydids differently. Sympatric predators were more likely to attack, kill and eat mountain katydids compared to allopatric predators. We see two potential, non-mutually exclusive explanations for this pattern: (1) the behaviour of the predators reflects regional variation, (2) predator experience influences the efficacy of the display. Differences in sympatric and allopatric magpie behavior may reflect regional variation in predator ecology, such as local adaptation, poorer condition or less discerning sympatric predators, or heightened neophobia and/or wariness among allopatric predators^39,41,42^. These explanations are unlikely, however, as ample alternative food sources were available during the time we conducted the experiment, late summer, and within the wide range of both sympatric and allopatric magpies^43^. There is a considerable diversity of aposematic invertebrates in the range of our allopatric magpies, which would offer opportunity for experience with defended prey, for example the transverse ladybird, *Coccinella transversalis*. However, the distinctiveness of the mountain katydids’ appearance^44^ minimises any potential for Batesian mimicry to provide an explanation for our results. We do acknowledge, however, that while individual magpies were drawn from a large geographic area relative to their known territory size (and thus represent several distinct populations) it would be valuable to extend this experiment using entirely independent predators across a more closely controlled range of experience, to further examine the above explanations.

Our results are consistent with two mechanisms by which the mountain katydid’s display provides protection; deimatism and aposematism^1,11,17,24^. Below, we discuss both possibilities, and a third, that the defense has both deimatic and aposematic components, making it a multi-component display.

Upon first presentation of a katydid, naïve allopatric predators were less likely to attack, kill and eat katydids than experienced sympatric predators. Recent suggestions predict that deimatism should be effective against naïve predators because it is thought to exploit ‘hard-wired’ reflexive responses that do not require learning^11,14,17^. Deimatism is not the only mechanism by which prey might be protected from naïve predators, since initial avoidance can also be facilitated by forms of neophobia and dietary conservatism^1^. However, under these mechanisms, one may not expect the predator to approach and attack novel prey as they did in our study, reducing the likelihood of this hypothesis in our case. Nevertheless, protection by their display against naïve predators is not predicted under aposematism, which is usually described as a learned signal^1^.

Allopatric predators reduced their attack rate markedly in the second encounter compared to the first. This is expected under aposematism, where predators are less likely to attack after an adverse experience, however, this is not necessarily inconsistent with deimatism. In principle being ‘startled’ might be aversive enough for predators to avoid future interactions, especially given the relatively short temporal window between presentations in our experiment (2 min), which could have meant that a startle effect still lingered in the predator’s mind upon the second encounter. Whether this occurs in nature is currently unclear because there is little evidence as to whether deimatism enhances or inhibits learning. If we assume startle can be learned, it is unclear whether predators learn to pay attention to it or learn to ignore it. Dookie *et al*.^27^ showed declining protective value of auditory startle with repeated interactions between red-winged blackbirds (*Agelaius phoeniceus*) preying on walnut sphinx caterpillars (*Amorpha juglandis*)^27^ suggesting that predators learn to ignore the startle, but whether their result is generalisable across modes and taxa remains to be seen.

If the katydid’s defence is a combination of deimatism and aposematism, it is possible that during their first encounter in which the magpies attacked the katydids and were startled, the magpies tasted the katydids, saw their display and learned to associate those experiences with the rest (putative camouflage) phase, thus reinforcing aposematism. Therefore, the allopatric predator response can be explained if we consider that the katydid’s defence has multiple components: in the first instance the display deters predators based on reflexive recoil, but it also reinforces the association between bad taste and colorful signal^16^, reducing the likelihood of a subsequent approach.

The reduced effectiveness of the katydid’s display against sympatric, experienced predators (Fig. 2c), is noteworthy and is consistent with theory on deimatism being the sudden transitional element that can lead from camouflage to aposematism^17^. There are several non-exclusive facets to this explanation. One is that sympatric, experienced predators may have learned to suppress their reflexive aversion to the deimatic component of the katydid’s display, at least in part, by expecting it. The circumstances or number of trials this might take is currently unknown. Our study is limited to two interactions, further experiments that aim to tease apart aposematism and deimatism may need to test predator responses in a series of interactions. This is consistent with work on vertebrate predators showing that, while highly sensitive to movement in general, they may rapidly learn to disregard non-salient motion cues following repeated exposure^45–47^. We further suggest that the katydid’s display reveals an aposematic signal, but that sympatric, experienced predators have behavioural or physiological strategies to resist chemical defences or they may be able to consume chemically defended prey by making strategic decisions about when to incur the assumed cost of consuming them^26,40,48^. Two observations of Australian magpies living sympatrically with mountain katydids suggest that at least some individuals wash^31^ and wipe (Umbers and De Bona pers. obs.) them before consumption which may imply that sequestered compounds are stored in secretions rather than body tissue. Another possibility is that the mountain katydids’ chemical-defence is less distasteful or toxic than has been suggested making them a relatively undefended deimatic displayer^28^. Their diet of alkaloid-rich *Senecio*, and the strong aversive response of naïve predators upon secondary presentation (Fig. 2c, orange dashed line), suggest a level of chemical protection, though this question remains to be explored in detail.

Many features of deimatic displays seem counter-intuitive. For example some species, such as the mountain katydid, wait until they have been physically attacked before performing their display^30^. This is difficult to explain because theory suggests that deimatic displays should be performed as the predator approaches, causing it to stop or at least pause its attack^11,24^. Considering our results, however, we suspect that mountain katydids rely on camouflage when possible, because their natural, sympatric predators seem to have learned to attack and consume them despite their complex defence. Moreover, most predator communities contain at least some proportion of naïve predators (e.g. fledglings). Thus, despite any chemical defence, the best strategy for many prey is not to be overtly conspicuous^49,50^ or perform their display too often, if its efficacy is partly based on predator inexperience.

Despite a growing appreciation of the importance of defensive displays^15,18,30,51,52^, unravelling the mechanisms by which they confer protection, and how they evolve, remains a challenge^17^. Teasing apart the various components of defensive displays and understanding how they work in concert is a major challenge for this field. Among predators, innate aversive responses to threatening stimuli are ubiquitous, and arise early in development^19,53–57^. Such perceptual biases are well known to serve as the substrate for signal evolution, though this has chiefly been explored in the context of sexual communication^58,59^. Male field crickets (Eneopterinae: Lecithin), for example, seemingly exploit the startle-responses of females by generating high frequency calls that are typically associated with local predators^60^. These calls goad females into producing a vibrational response, which males can then use to locate them. Similarly, our results support the notion that the existence of reflexive biases in predators can generate “evolutionary opportunities” for exploitation in defensive strategies (Fig. 2c). These opportunities may even underlie the initial evolution of deimatism, which could arise through the inadvertent exploitation of reflexive responses (e.g. through pre-escape-flight movements) before being elaborated into a dedicated display^14,17,23^. This stands in contrast to aposematic systems which, owing to their reliance on general learning rules in predators, are thought to require the evolution of prey defences among cryptic ancestors prior to conspicuous signals^1,9,61^. However, this does not mean that deimatism and aposematism are mutually exclusive, we expect that in many species they could work in concert. Ultimately, our results offer some early empirical support for the protective value of a complex defensive display and provide a strong field-based system with which to understand the mechanistic side of these charismatic defences.

### Ethical note

This experiment was conducted under ethics permit AE14/35.

## Supplementary information

Supplementary Dataset 1

Supplementary Video S1

## Data Availability

Data will be made available on figshare.
